# Early Intravenous Beta-Blockade with Esmolol in Adults with Severe Traumatic Brain Injury: A Phase 2a Intervention Design Study

**DOI:** 10.1007/s12028-024-02029-8

**Published:** 2024-06-28

**Authors:** Matt Thomas, Kati Hayes, Paul White, Thomas Baumer, Clodagh Beattie, Aravind Ramesh, Lucy Culliford, Gareth L. Ackland, Anthony E. Pickering

**Affiliations:** 1 Intensive Care Unit, North Bristol NHS Trust, Bristol, UK; 2 Research and Development, North Bristol NHS Trust, Bristol, UK; 3 School of Data Science and Mathematics, University of the West of England, Bristol, UK; 4 Severn Deanery, Bristol, UK; 5 Faculty of Health Sciences, University of Bristol, Bristol, UK; 6 Bristol Medical School (PHS), Bristol Trials Centre, University of Bristol, Bristol, UK; 7 William Harvey Research Institute, Queen Mary University of London, London, UK; 8 School of Physiology, Pharmacology and Neuroscience, University of Bristol, Bristol, UK

**Keywords:** Traumatic brain injuries, Adrenergic beta-antagonists, Intensive Care Unit, Adaptive clinical trial

## Abstract

**Background:**

Targeted beta-blockade after severe traumatic brain injury may reduce secondary brain injury by attenuating the sympathoadrenal response. The potential role and optimal dosage for esmolol, a selective, short-acting, titratable beta-1 beta-blocker, as a safe, putative early therapy after major traumatic brain injury has not been assessed.

**Methods:**

We conducted a single-center, open-label dose-finding study using an adaptive model-based design. Adults (18 years or older) with severe traumatic brain injury and intracranial pressure monitoring received esmolol within 24 h of injury to reduce their heart rate by 15% from baseline of the preceding 4 h while ensuring cerebral perfusion pressure was maintained above 60 mm Hg. In cohorts of three, the starting dosage and dosage increments were escalated according to a prespecified plan in the absence of dose-limiting toxicity. Dose-limiting toxicity was defined as failure to maintain cerebral perfusion pressure, triggering cessation of esmolol infusion. The primary outcome was the maximum tolerated dosage schedule of esmolol, defined as that associated with less than 10% probability of dose-limiting toxicity. Secondary outcomes include 6-month mortality and 6-month extended Glasgow Outcome Scale score.

**Results:**

Sixteen patients (6 [37.5%] female patients; mean age 36 years [standard deviation 13 years]) with a median Glasgow Coma Scale score of 6.5 (interquartile range 5–7) received esmolol. The optimal starting dosage of esmolol was 10 μg/kg/min, with increments every 30 min of 5 μg/kg/min, as it was the highest dosage with less than 10% estimated probability of dose-limiting toxicity (7%). All-cause mortality was 12.5% at 6 months (corresponding to a standardized mortality ratio of 0.63). One dose-limiting toxicity event and no serious adverse hemodynamic effects were seen.

**Conclusions:**

Esmolol administration, titrated to a heart rate reduction of 15%, is feasible within 24 h of severe traumatic brain injury. The probability of dose-limiting toxicity requiring withdrawal of esmolol when using the optimized schedule is low.

*Trial registrationI* SRCTN, ISRCTN11038397, registered retrospectively January 7, 2021 (https://www.isrctn.com/ISRCTN11038397).

**Supplementary Information:**

The online version contains supplementary material available at 10.1007/s12028-024-02029-8.

## Introduction

Severe traumatic brain injury (TBI) is devastating for patients and families and imposes a significant burden on society. The primary injury, suffered at impact, cannot be altered by medical intervention. Secondary injury, developing minutes, hours, and days after the event, can be improved by medical treatment. Mitigation of secondary injury should reduce death, disability, and economic cost after TBI. Current mitigation strategies are conservative, emphasizing physiological stability and prevention or early treatment of complications such as seizures or intracranial hypertension. Additional neuroprotective interventions remain as yet unproven [1–5].

The stress response to trauma includes activation of the sympathetic nervous system and adrenal glands, producing a surge in circulating catecholamines to support blood pressure and cardiac output [6]. However high and/or prolonged levels of circulating catecholamines can have adverse effects. The shock-induced endotheliopathy paradigm holds that these high levels of catecholamines damage vascular endothelium, resulting in increased vascular permeability, thrombosis, and inflammation [7]. In the cerebral circulation, damage predisposes to the development of edema, neuroinflammation, and possibly impaired neurovascular regulation, all of which contribute to secondary brain injury [8].

In TBI, plasma catecholamine levels correlate with endothelial injury, inflammation, neurological deficit, and outcome [9–11]. In animals, beta-blockers after TBI reduce cerebral edema, preserve autoregulation, and are associated with improved recovery [12–14]. Prospective studies in humans are supportive but are relatively small, using heterogenous regimens of drugs, doses, durations, and combinations, and have limited external validity [15–20]. Meta-analyses show a reduction in mortality and improvement in long-term functional outcomes at the cost of longer hospital stays and increased infectious and cardiopulmonary complications [21–26].

Having been in use since the 1960s, beta-blockers are familiar drugs in intensive care with well-described actions and side effects. They are low cost and have widespread availability. Esmolol specifically is titratable, combining rapid onset of action (and potential neuroprotection) with rapid offset (if side effects occur) [27]. This potentially enables early use within hours of trauma, balancing possible benefit against the risk of compromising physiological stability.

In addition, esmolol has been shown to be protective in animals after brain or spinal cord ischemia [28, 29]. In humans, it reduces inflammation after surgical trauma, provides cerebral cortical electrical suppression in the context of anesthesia, and does not impair cerebral blood flow in volunteers or during electroconvulsive therapy [30–33]. It is associated with lower intracranial pressure (ICP) if used in the first 24 h after TBI [20]. In severe TBI, beta-1 selective blockers, such as esmolol, have been shown to not alter cerebral blood flow [34]. These effects are all potentially neuroprotective.

There is no consensus on the optimal beta-blocker treatment schedule for TBI [24]. Working from the hypothesis that beta-blockade improves mortality and morbidity after severe TBI, the Early Intravenous Beta-Blockade with Esmolol in Adults with Severe Traumatic Brain Injury (EBB-TBI) program aims to define and test a standardized approach. This study is the first step, which, given the uncertainty around benefit and the importance of maintaining cerebral perfusion pressure, is designed to determine a safe dosage for early beta-blockade in adults after severe TBI.

## Methods

### Study Design

EBB-TBI is a single-arm, open-label dose-finding study of esmolol for adults with severe TBI that uses the continual reassessment method, an adaptive model-based design [35, 36]. The setting is a single mixed intensive care unit (ICU) in a major trauma center in South West England serving an adult population of approximately 2.3 million. The full protocol is available in the Supplementary Material, with additional background and extended discussion accompanying presentation of the study design, methods, and intervention published elsewhere as the trial protocol [37].

The study was approved by South Central—Hampshire A Research Ethics Committee (reference 20/SC/0219). The study was registered on January 7, 2021, with International Standard Randomised Controlled Trial Number 110383897 (https://www.isrctn.com/ISRCTN11038397).

### Population

Adult patients (aged ≥ 18 years) were eligible if the time of assessment for study entry was within 24 h of head injury, if they had a Glasgow Coma Scale (GCS) score of 8 or less after resuscitation or prior to intubation, and if they had ICP monitoring in situ. Major exclusion criteria were a life-threatening or limb-threatening extracranial injury, a devastating brain injury, or a contraindication to the use of beta-blockade. A full list is given in the protocol. A deferred model of consent was used, as patients lacked capacity given the severity of injury, and there was a need for early intervention.

### Intervention

Patients received open-label esmolol (Brevibloc Premixed 10 mg/mL solution for infusion; Baxter Healthcare Ltd) started within 2 h of confirmation of eligibility. The initial starting dosage (dosage level 1, Table 1) was based on a dosage regimen shown to be tolerated by a group of mechanically ventilated, vasopressor-dependent patients with septic shock [38]. Esmolol was titrated to the heart rate as a convenient clinical biomarker of the stress response, with the target set as a 15% reduction from baseline (the average heart rate in the 4 h preceding confirmation of eligibility for study enrollment) [37]. Actual body weight was used for dosage calculations, with a maximum permitted dosage of 200 μg/kg/min.

**Table 1:** Predefined dosage levels for esmolol infusion

Dosage level (*d*_*n*_)	Start dosage (μg/kg/min)	Increment dosage (μg/kg/min)
1	5	2.5
2	10	5
3	16	8
4	25	12.5
5	35	17.5
6	46	23
7	62	31

Each cohort was treated at a single dosage level

*d*_*n*_, dosage level *n*

The infusion was continued until one of the following stopping rules was met:

1. Ninety-six hours from start of infusion
2. Heart rate target achieved without esmolol for > 12 h
3. Failure to meet cerebral perfusion pressure (defined as mean arterial pressure measured at the tragus minus ICP) target of 60–70 mm Hg for > 4 h
4. Serious adverse event attributable to esmolol
5. Death or withdrawal of life-sustaining treatment
6. Request of participant, legal representative, or responsible consultant

No other selective or nonselective beta-blockers were permitted during the esmolol intervention phase; after this time, use was at the discretion of the clinical team. All other treatment was directed by the clinical team following usual local practice based on Brain Trauma Foundation guidelines [1].

### Outcomes

The primary outcome was a maximum tolerated dosage escalation schedule of esmolol that combined a clinically significant reduction in heart rate (defined as ≥ 15% from baseline) with maintenance of cerebral perfusion pressure. Secondary outcomes were the following: Sequential Organ Failure Assessment (SOFA; excluding neurological assessment; daily during intervention) score; ICU, hospital, and 6-month mortality; ICU and acute hospital length of stay; duration of mechanical ventilation; bloodstream infection in ICU; 6-month extended Glasgow Outcome Scale; and 6-month quality of life (EQ-5D-5L).

Exploratory outcomes assessed during the intervention included laboratory biomarkers (including cardiac troponin T, glucose, and lactate levels), incidence of bradycardia, heart block or clinically significant hypotension (defined as blood pressure below Brain Trauma Foundation recommendations), vasopressor dose, proportion of time with cerebral perfusion pressure in range, and interventions per day for ICP control (quantified as summary and domain therapy intensity levels).

### Statistical Analysis

The sample size of up to 24 participants was determined pragmatically based on the expected information yield for a sample size in the modified continual reassessment method (CRM) study design with a cohort size of 3. Dose levels (*d*_1_–*d*_7_) are shown in Table 1, with the decision to move between dosage levels made by the study management group after each cohort of participants had been assessed for dose-limiting toxicity. In the absence of dose-limiting toxicity, the subsequent cohort of participants was treated at the next dosage level (subject to the additional safety measure being no permitted escalation between the first and second cohorts). For example, in the absence of dose-limiting toxicity at dosage level 1 (*d*_1_) in cohort two (participants 4–6), then cohort three would be treated at dosage level 2 (*d*_2_).

Other modifications to the CRM design for this study are listed in the protocol in the Supplementary Material. A one-parameter logistic model initialized with skeleton parameters as per Table 1 was used for the CRM modeling, with estimated probabilities revised as data emerged. This likelihood modeling algorithm will identify a maximum tolerated dosage with a defined prior reasoned target toxicity level (or “acceptable” toxicity rate, θ) of 10% with an indifference level of two percentage points for decision-making.

Continuous variables are summarized by descriptive statistics (mean and standard deviation [SD], minimum, median, and maximum and interquartile range [IQR]) and categorical data in terms of frequency and percentage. There are no subgroup or adjusted analyses.

## Results

**Fig. 1:**
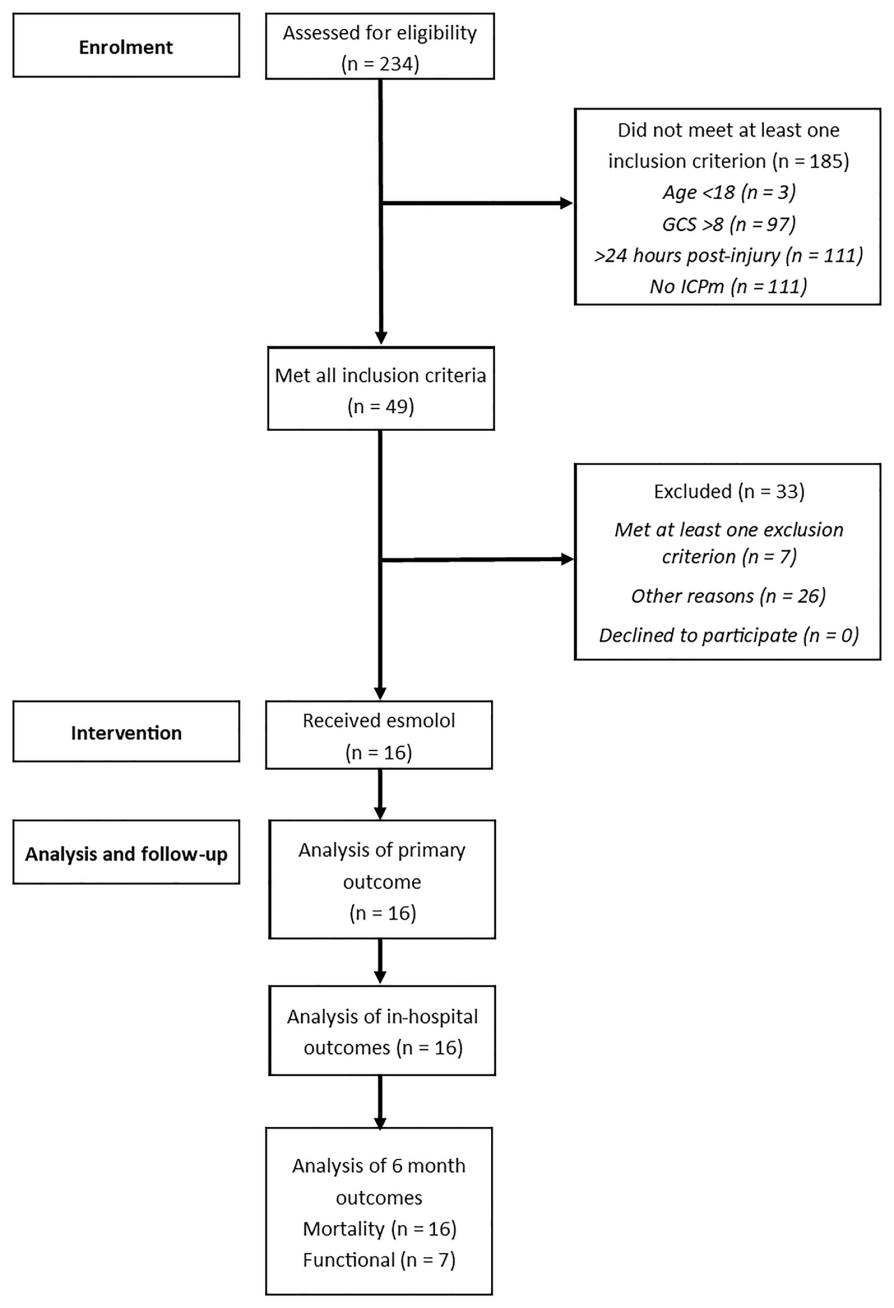
Enrollment, intervention, analysis, and follow-up of participants. Participants may not meet more than one inclusion criterion. GCS Glasgow Coma Scale, ICPm intracranial pressure monitor

The study opened to recruitment on November 30, 2020, and completed follow-up on April 21, 2023, having exceeded funded time and with agreement of the sponsor that the primary research question could be answered; 234 patients were assessed for eligibility, with 16 patients receiving esmolol (Fig. 1). At the time of screening, a total of 49 patients met all inclusion criteria, with 7 of these meeting at least one predefined exclusion criterion (most frequently a perceived devastating brain injury, *n* = 4). Other reasons for exclusion were a baseline heart rate below the minimum permitted target (60 beats per minute, *n* = 4), lack of medically trained staff to confirm eligibility (*n* = 1), and admission during an unscheduled pause in recruitment (due to either COVID-19 surge, dosage escalation review, or clinical information system safety testing). The predominant reasons for not meeting inclusion criteria were absence of ICP monitoring or screening more than 24 h after injury.

Baseline characteristics are shown in Table 2. Of 16 patients, 10 were male and 6 were female, and the mean age was 36.3 years (SD 13.0 years). The median GCS score at presentation was 6.5 (IQR 5–7), and half of the patients had isolated TBI. The median Charlson Comorbidity Index was 0, and no patients had a history of beta-blocker use prior to injury.

**Table 2:** Baseline characteristics (*n* = 16)

	Value
Age (years)	
Mean (SD)	36.25 (13.03)
Median (IQR)	33.5 (26.5–47.5)
Sex, *n*	
Male	10
Female	6
Weight (kg)	
Mean (SD)	76.0 (16.78)
Median (IQR)	74.0 (62.5–87.5)
Charlson Comorbidity Index, median (IQR)	0 (0–0)
Mechanism of injury, *n*	
Fall (< 2 m)	2
Fall (> 2 m)	4
Road traffic collision	6
Other^a^	4
Initial cranial CT findings,^b^ *n*	
Extradural	3
Subdural	8
Subarachnoid	9
Parenchymal	9
Diffuse axonal injury	1
Skull fracture	8
GCS at presentation, median (IQR)	6.5 (5–7)
Helsinki CT score, median (IQR)	3.5 (2–5)
APACHE II, median (IQR)	11.5 (7.5–15)
Injury severity score, median (IQR)	29.5 (25.25–38)
Isolated TBI,^c^ *n* (%)	8 (50)
Preinjury beta-blocker use,* n* (%)	0 (0)

*AIS* Abbreviated Injury Scale, *APACHE* Acute Physiology and Chronic Health Evaluation, *CT* Computed tomography, *GCS* Glasgow Coma Scale, *IQR* interquartile range, *SD* standard deviation, *TBI* traumatic brain injury

^a^Fall from horse (*n* = 2), fall from motocross bike (*n* = 1), hit by train (*n* = 1)

^b^May total more than 16, as initial imaging may show more than one major intracranial injury

^c^Isolated TBI is head AIS score ≥ 3 and all other AIS scores of < 3

The median time from injury to commencement of esmolol was 20.7 h (IQR 16.5–23.9 h; Table 3). Esmolol was given for a mean of 60.1 h (SD 41.1 h) of the 96-h intervention period at an overall mean dosage of 69.2 μg/kg/min (SD 61.6 μg/kg/min).

**Table 3:** Additional baseline characteristics

	Value
Injury to emergency department (hours), median (IQR)	0.4 (0.2–0.56)
Injury to hospital admission (hours), median (IQR)	1.9 (1.7–2.1)
Injury to confirmation of eligibility (hours), median (IQR)	18.9 (15.0–21.5)
Injury to commencement of esmolol (hours), median (IQR)	20.8 (16.5–23.9)
Heart rate at commencement of esmolol (bpm), mean (SD)	82.13 (15.25)
Heart rate at commencement of esmolol (bpm), median (IQR)	79.3 (70.3–90.3)

*Bpm* beats per minute, *IQR* interquartile range, *SD* standard deviation

**Fig. 2:**
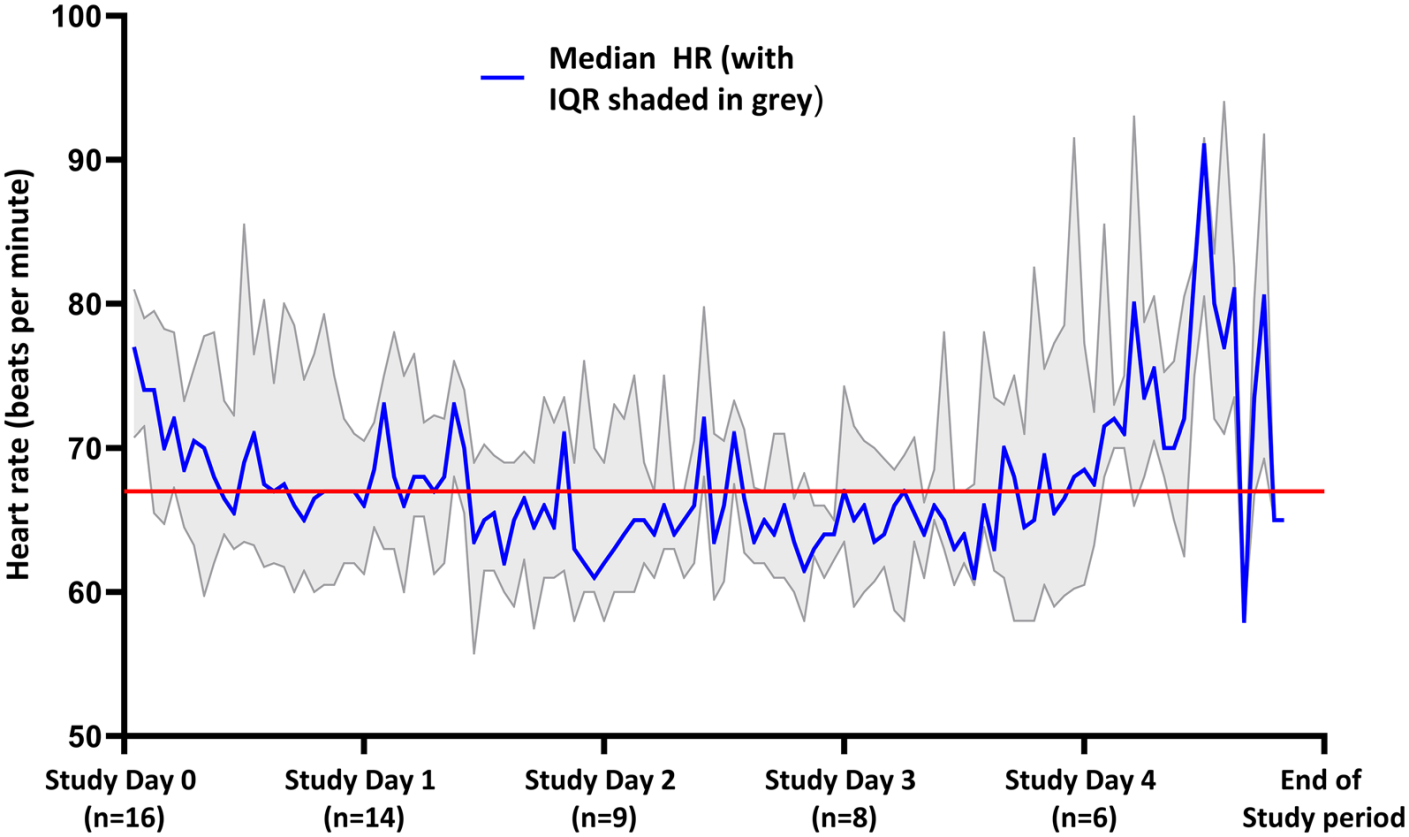
HR control during esmolol intervention period. Red line shows the median target heart rate. HR heart rate, IQR interquartile range

The median baseline heart rate prior to infusion was 79.3 beats per minute (IQR 70.3–90.3 beats per minute). The heart rate during intervention was well controlled, meeting the target of 15% reduction from baseline (Fig. 2).

### Primary Outcome

**Fig. 3:**
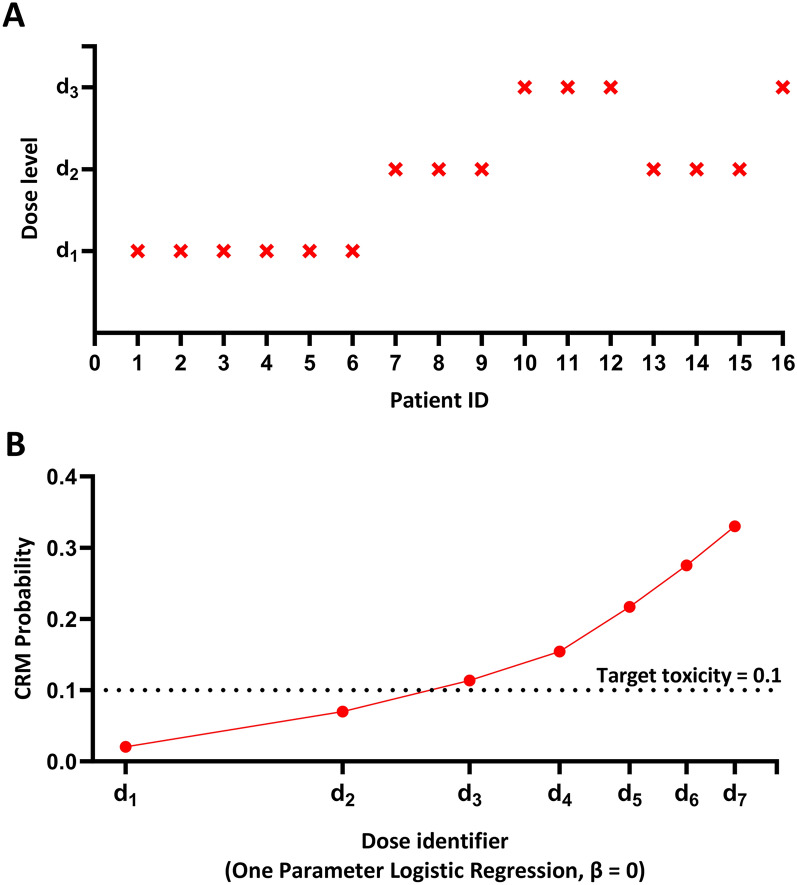
Dose levels and estimated probability of dose-limiting toxicity. The estimated probabilities of dose-limiting toxicity were 0.07 with use of dosage level 2 and 0.11 with dosage level 3, which falls above the predefined acceptable probability of toxicity (0.1). CRM continual reassessment method, d_n_ dosage level n, ID identifier

Six patients were treated at dosage level 1, followed by three patients at dosage level 2 and a further three patients at dosage level 3. After identification of a dose-limiting toxicity event (cerebral perfusion pressure of 59 mm Hg for two consecutive hours and then 58 mm Hg for two further consecutive hours) the next three patients were treated at dosage level 2, followed by one patient at dosage level 3 (Fig. 3).

The estimated probability of dose-limiting toxicity at each prespecified dosage level is shown in Fig. 3. Dosage level 2 (starting at 10 μg/kg/min, with increments of 5 μg/kg/min) had a probability of dose-limiting toxicity of 0.07, meeting the a priori criterion for an acceptable level of toxicity.

### Secondary Outcomes

All-cause 6-month mortality was 12.5% (Table 4) against an expected 20% based on Helsinki computed tomography (CT) scores, giving a standardized mortality ratio of 0.63 (95% confidence interval 0.45–0.80). At six months, the median extended Glasgow Outcome Scale score was 4 (IQR 2.5–4.5, *n* = 7) and the median EQ-5D-5L visual analog scale was 62.5 (IQR 55–65, *n* = 5).

**Table 4:** Secondary outcomes (*n* = 16)

	Value
Mortality (ICU), *n* (%)	2 (12.5)
Mortality (acute hospital),* n* (%)	2 (12.5)
Mortality (6 months),* n* (%)	2 (12.5)
Duration of mechanical ventilation (days), median (IQR)	13.5 (8.8–17)
Length of ICU stay (days), median (IQR)	18.5 (12.8–22.3)
Length of acute hospital stay (days), median (IQR)	26 (22.3–48.8)
Bloodstream infection in ICU,* n* (%)	0 (0)

*ICU* intensive care unit, *IQR* interquartile range

Clinical interventions, including vasopressor use, cerebral perfusion pressure maintenance, and management of intracranial hypertension, are shown in Supplementary Tables 1 and 2. Nearly all patients required vasopressor support with either metaraminol or noradrenaline for maintenance of cerebral perfusion pressure, which was above the minimum (60 mm Hg) for 90% of the intervention period. Clinical management of intracranial hypertension is reflected by the therapy intensity level, showing relatively few interventions required after the first study day beyond positioning and sedation.

## Discussion

In adults, administration of esmolol within 24 h of severe TBI at a starting dosage of 10 μg/kg/min with increments of 5 μg/kg/min titrated to a heart rate reduction of 15% from baseline is associated with a low probability (0.07) of dose-limiting toxicity necessitating withdrawal of esmolol. The infrequent hemodynamic adverse effects can be managed using standard vasopressor titration with cerebral perfusion pressure defended for 90% of the infusion time. An internal review of the single dose-limiting toxicity event suggested that this risk could be minimized with consistent adherence to study flowcharts. Defining toxicity using a single metric, although necessary for the CRM, is an oversimplification of the complex effects of beta-blockade in critically ill patients. Nevertheless, our findings support the safety and feasibility of early beta-blockade with esmolol within 24 h of severe TBI in adults.

The observed 6-month mortality of 12.5% is less than that predicted by the Helsinki CT score in our cohort [39, 40]. It is also lower than the ICU stratum of Collaborative European NeuroTrauma Effectiveness Research in TBI (CENTER-TBI; 15% in a cohort of patients with both moderate and severe TBI) and lower than that shown in the Trauma Audit and Research Network analysis of TBI in England (40.4% for severe TBI) [41, 42]. In the absence of a control group, we are unable to attribute this to esmolol. It may reflect study bias or nonprotocolized aspects of care in our center. It is however consistent with findings in a meta-analysis of beta-blockade in TBI and supportive of further research to investigate a potential benefit of esmolol.

This is only the third study reporting the use of esmolol in adults after severe TBI. In an observational study in Iran, 12 patients exposed to a loading dose (0.5 mg/kg) and then a 24-h fixed dosage infusion of esmolol (50 mg/kg/min) had lower ICP during that period than a contemporary control group, with no differences in heart rate or mean arterial pressure [20]. We have shown that a longer duration of infusion is feasible, as is dosage adjustment to heart rate as a simple biomarker of the stress response. Our exclusion criteria are less restrictive, and our starting point is better defined, which, together with the difference in clinical setting, extends the generalizability of the intervention.

In a larger observational study from the United States, esmolol use was reported in 7% of the 1120 patients (from a cohort of 2337) receiving any beta-blocker for any usual indication rather than for neuroprotection during an ICU stay. Only 38% of patients had severe head injury (GCS score ≤ 8). No esmolol-specific outcomes were reported, although both beta-1 selective and all beta-blocker use were associated with reduced 30-day mortality [43]. Our study adds data on longer-term mortality associated with esmolol in a more severely injured cohort.

Early administration of beta-blockade with titration to heart rate was tested in a randomized controlled trial of metoprolol, another beta-1 selective beta-blocker, in 60 patients in Egypt [18]. In this study, reductions in mortality (seen in patients > 40 years old only) and ICU length of stay and improvement in Glasgow Outcome Scale scores at 1 month were shown in the intervention group. Outcomes were better in those achieving the fixed heart rate target of 60–70 beats per minute early, though this was not further defined. Dose titration was made on a 6-h cycle. Baseline heart rates, time taken to achieve target, and other hemodynamic data are not available in the study report. We have shown that use of an intravenous drug with titration every 30 min allows rapid target attainment. Whether this translates into greater benefit and whether a relative or an absolute heart rate target is preferable requires further research.

As with these prior studies, we chose not to protocolize all aspects of management, particularly those such as analgesia and sedation or fluid resuscitation that could have a significant bearing on heart rate early after major trauma. Indeed, given the reducing number of patients requiring esmolol each study day, it is likely other factors, such as the natural time course of the stress response, were contributing to heart rate reduction over time. The advantages of this protocol approach are simplicity in terms of intervention delivery—there is only one infusion and one target—and pragmatism when scaling up to effectiveness trials. The disadvantages are somewhat mitigated by consistent patient management practice in a single center. A key next step in the EBB-TBI program is the trial of the intervention at more sites.

Another consideration for future research is the wide interindividual variability in the patient population in terms of stress response (evidenced by baseline heart rate), dosage of esmolol, and duration of infusion. This likely reflects the heterogeneity of the TBI population, arising from patient, mechanism, intracranial and extracranial injury pattern, and interventions received. In future work, we plan to undertake assessment of biomarkers, including of brain, endothelial, immune, and sympathetic function, to try to define subphenotypes for subsequent study enrichment. Cardiac troponin remains an attractive and simple biomarker that could define a group most likely to benefit [44]. In its absence, titration of esmolol to a proportional heart rate reduction as a biomarker of pharmacodynamic engagement indicating a level of beta-blockade that might be sufficient to provide neuroprotection seems a rational approach. The simplicity and universal availability of heart rate balances the fact that it is at best a crude approximation of the stress response. Our target of a 15% reduction from baseline is convenient and may be safe but is not proven to be the ideal sole marker for posttraumatic neuroprotection [37]. Whether any particular modality of advanced neuromonitoring would add to safety (by identifying low cerebral blood flow or suboptimal perfusion pressure, for example) or to effect (by identifying groups likely to benefit) is as yet unknown.

We acknowledge the apparent contradiction in use of beta-blockade with concurrent use of catecholaminergic vasopressors. The resulting in vivo balance between alpha-1, beta-1, and beta-2 activation and the consequent effect on pathophysiology is difficult to predict. The stable SOFA scores and lactate values we observed argue for the maintenance of hemodynamics and vital organ perfusion. In terms of secondary brain injury, there is evidence to show that phenylephrine (a pure peripheral alpha-agonist) is associated with lower in-hospital mortality after severe TBI [45]. This is consistent with harm from excessive catecholamines being mediated via beta-receptors, which may potentially be mitigated with beta-blockade.

Propranolol is a nonselective beta-blocker that is frequently used after TBI and favored by some, but not all, as the first-line neuroprotective beta-blocker [16, 18, 43, 46, 47]. Prospective randomized trials comparing selective against nonselective beta-blockers in this setting have not been undertaken. A previous review favoring propranolol uses a heterogenous comparator of all other selective and nonselective beta-blockers together with those with additional actions (e.g., labetalol, sotalol) [43]. In our opinion, the theoretical and practical advantages of esmolol make it deserving of further research, including potentially in direct comparison with propranolol in the ICU setting.

There are still other potential strategies (such as dexmedetomidine, a centrally acting alpha-agonist that reduces sympathetic outflow) that could mitigate harm associated with excess catecholamines [48]. Whichever strategy is chosen, underlying mechanisms are complex and extend beyond simple hemodynamics to regulation of fundamental metabolic, immune, and inflammatory pathways [49, 50]. Further research is needed to determine the ideal therapeutic approach.

The strengths of our study include the pragmatic approach, broad inclusion criteria (especially compared to other trials of beta-blockade in TBI) [15, 17–19], and the application of an established methodology to a novel setting and research question. Our study also has limitations. It represents practice in a single center, so intervention delivery and effect may not generalize to other units. A high proportion of the patients admitted with TBI did not meet the inclusion criteria, further limiting generalizability. In part, this reflects local practice in patient selection for ICP monitoring. In addition, a lack of out-of-hours research staff contributed to the number of patients excluded because they were identified more than 24 h after injury. This also prevents deeper analysis of the patient population to try to identify subgroups more likely to receive benefit or suffer harm. We cannot differentiate the relative contribution of esmolol and other interventions (such as fluid or sedation) on heart rate control or clinical outcomes. We chose a conservative intervention period focused on the point of greatest physiological instability and prior to the typical development of cerebral edema and cannot determine whether this is the ideal duration of infusion. Our sample is small, meaning there is potential for uncertainty in our estimate of maximum tolerated dosage. This is mitigated by titration to effect for each patient. We had a high loss to longer-term follow-up, perhaps relating to the large catchment area and cognitive impairment or psychological distress in survivors, although this did not compromise analysis of the primary outcome.

This study adds to the small number of esmolol-treated patients with severe TBI reported in the literature. Our results are consistent with the positive findings of other studies with esmolol or alternative beta-1 selective blockers, which supports the argument for further research into early beta-1 selective blockade. We have identified some key areas of uncertainty, among which we consider the need to identify severe TBI populations most likely to benefit, the choice of beta-1 or nonselective beta-blockade, and the influence of other interventions (such as sedation or surgery), as the most important. Given these uncertainties and the very small number of esmolol-treated patients (28, including in this study) in only two centers worldwide with surrogate primary outcomes, it is too early to recommend esmolol for widespread use or even for a randomized trial of effectiveness. Further study in multiple centers and larger cohorts with analysis of a range of biomarkers reflecting both the complexity of the stress response and severity of cranial and noncranial injury is necessary, along with data on relevant patient-centered outcomes [51]. With better understanding of pathophysiology and the links to outcomes, better therapy can be identified and personalized [52].

## Conclusions

We have shown that early beta-blockade with esmolol in adults after severe TBI is feasible and may be associated with a mortality benefit. We have determined a treatment schedule that can be tested for patient benefit in future trials.

## Supplementary Information

Below is the link to the electronic supplementary material.

Supplementary file1 (DOCX 23 kb)

Supplementary file2 (DOCX 21 kb)
