# Supplementary material for: Early Intravenous Beta-Blockade with Esmolol in Adults with Severe Traumatic Brain Injury: A Phase 2a Intervention Design Study
Source: Neurocrit Care. 2024 Jun 28;41(3):1009–19. doi: 10.1007/s12028-024-02029-8 (PMC11599627; doi:10.1007/s12028-024-02029-8)
Supplement: Supplementary file 1 — Supplementary file1 (DOCX 23 kb) [file 12028_2024_2029_MOESM1_ESM.docx]

| Secondary outcomes – organ function and exploratory outcomes (n=16) | | | | | | |
| --- | --- | --- | --- | --- | --- | --- |
|  | Study day 0 | Study day 1 | Study day 2 | Study day 3 | Study day 4 | Study days 0 – 4 |
| 1. Organ function – SOFA score* | | | | | | |
| *Patients receiving esmolol (/n)* | *16* | *14* | *9* | *8* | *6* | *53* |
| Modified SOFA score daily [/20]* (median/IQR) | 6.0 (4.8 – 7.5) | 7.0 (6.0 – 8.8) | 5.0 (5.0 – 6.0) | 6.0 (5.0 – 7.0) | 6.5 (5.6 – 7.8) | 6.0 (5.0 – 7.0) |
| 3a. Biomarkers | | | | | | |
| *Patients troponin measured (/n)* | *11* | *14* | *9* | *8* | *6* | *48* |
| Troponin, ng/L  (median/IQR) | 9 (6 – 10) | 12 (7 – 22) | 6 (5 – 13) | 9 (7 – 10) | 6 (5 – 8) | 9 (6 – 13) |
| *Patients INR measured (/n)* | *15* | *14* | *8* | *7* | *4* | *43* |
| International normalised ratio, INR (median/IQR) | 1.0 (1.0 – 1.1) | 1.0 (1.0 – 1.0) | 1.0 (1.0 – 1.1) | 1.1 (1.0 – 1.1) | 1.0 (0.9 – 1.0) | 1.0 (1.0 – 1.1) |
| *Patients glucose/lactate measured (/n)* | *16* | *14* | *9* | *8* | *6* | *53* |
| Glucose low, mmol/L  (median/IQR) | 6.3 (5.7 – 6.6) | 6.1 (5.7 – 6.4) | 6.0 (5.6 – 6.4) | 6.3 (5.9 – 7.0) | 6.3 (5.4 – 7.3) | 6.1 (5.6 – 6.7) |
| Glucose high, mmol/L  (median/IQR) | 8.3 (6.9 – 9.8) | 8.9 (8.0 – 10.2) | 8.3 (7.7 – 9.6) | 9.9 (7.7 – 12.4) | 9.2 (7.4 – 11.1) | 8.6 (7.6 – 10.2) |
| Lactate, mmol/L  (median/IQR) | 1.8 (1.3 – 2.1) | 1.4 (1.0 – 2.1) | 1.2 (1.1 – 1.6) | 0.9 (0.7 – 1.1) | 1.1 (0.9 – 1.8) | 1.3 (1.0 – 1.9) |
| 3b. Haemodynamics | | | | | | |
| *Patients receiving esmolol (/n)* | *16* | *14* | *9* | *8* | *6* | *53* |
| Total esmolol infusion time  (hours per study day) | 262 | 249 | 215 | 156 | 92 | 195 |
| Mean hourly heart rate, bpm  (median/IQR) | 69 (65 – 74) | 67 (62 – 73) | 65 (62 – 70) | 65 (62 – 67) | 67 (66 – 78) | 67 (62 – 74) |
| Bradycardia (n/%):   - Episodes of HR <50bpm* per day   + with haemodynamic compromise  + requiring clinical intervention   - Incidence per study day - Incidence per hour of esmolol | 2 (12.5)  0 (0)  0 (0)  0.000  0.000 | 4 (26.7)  0 (0)  0 (0)  0.000  0.000 | 3 (37.5)  0 (0)  0 (0)  0.000  0.000 | 0 (0)  0 (0)  0 (0)  0.000  0.000 | 1 (20)  1 (2)  1 (2)  0.200  0.011 | 10 (19.3)  1 (0.4)  1 (0.4)  0.040  0.002 |
| 2^nd^/3^rd^ degree heart block (n/%) | 0 (0) | 0 (0) | 0 (0) | 0 (0) | 0 (0) | 0 (0) |
| 3b. Blood pressure | | | | | | |
| *Patients receiving esmolol (/n)* | *16* | *14* | *9* | *8* | *6* | *53* |
| MAP <70 mmHg at any point during study day (n/%) | 15 (93) | 14 (100) | 8 (89) | 7 (88) | 3 (50) | 47 (89) |
| Clinical significant hypotension at any point during study day* (n/%) | 5 (31) | 5 (36) | 3 (33) | 2 (25) | 2 (33) | 17 (32) |
| 3c. Vasopressors | | | | | | |
| *Patients receiving esmolol (/n)* | *16* | *14* | *9* | *8* | *6* | *53* |
| Vasopressor required during study days with esmolol infusion (n/%) | 15 (94) | 14 (100) | 8 (89) | 7 (87) | 5 (92) | 49 (92) |
| Metaraminol (n/%):  Mean rate* (median/IQR)    Mean maximum rate* (median/IQR) | 3 (19)  2.3 (1.9 – 2.7)  5.0 (3.3 – 5.5) | 3 (21)  3.8 (3.7 – 4.1)  7.0 (5.4 – 7.3) | 1 (11)  2.8 (2.8 – 2.8)  5.5 (5.5 – 5.5) | 1 (13)  1.2 (1.2 – 1.2)  1.2 (1.2 – 1.2) | 0 (0)  0 (0 – 0)  0 (0 – 0) | 8 (15)  3.2 (2.1 – 3.9)  5.3 (3.2 – 7.0) |
| Noradrenaline (n/%):  Mean rate* (median/IQR)  Mean maximum rate* (median/IQR) | 13 (81)  0.13 (0.05 – 0.25)  0.26 (0.15 – 0.39) | 12 (86)  0.17 (0.07 – 0.27)  0.31 (0.09 – 0.41) | 8 (89)  0.17 (0.08 – 0.32)  0.10 (0.06 – 0.22) | 6 (75)  0.18 (0.14 – 0.22)  0.27 (0.21 – 0.28) | 5 (83)  0.10 (0.08 – 0.15)  0.19 (0.16 – 0.19) | 8.8 (83)  0.15 (0.06 – 0.27)  0.22 (0.14 – 0.39) |
| 3c. Cerebral perfusion pressure (CPP) | | | | | | |
| *Patients receiving esmolol (/n)* | *16* | *14* | *9* | *8* | *6* | *53* |
| Total esmolol infusion time  (hours per study day) | 262 | 249 | 215 | 156 | 92 | 974 |
| Hourly CPP 60-70 mmHg during esmolol infusion period (n/%) | 127 (48) | 103 (41) | 95 (44) | 66 (42) | 36 (39) | 427 (44) |
| Hourly CPP ≥60mmHg during esmolol infusion period (n/%) | 219 (84) | 180 (72) | 161 (75) | 117 (75) | 83 (89) | 759 (78) |
| Hourly CPP <60mmHg during esmolol infusion period (n/%) | 25 (10) | 44 (18) | 18 (8) | 15 (10) | 4 (4) | 106 (11) |
| Hourly CPP during esmolol infusion period not recorded (n/%) | 18 (7) | 25 (10) | 36 (17) | 24 (15) | 6 (7) | 109 (12) |

***** “Modified” sequential organ failure assessment (SOFA) score to exclude neurological assessment – out of 20 instead of 24; HR <50bpm (heart rate <50 beats per minute); MAP (mean arterial pressure); Clinically significant hypotension – systolic blood pressure <100 mmHg for patients aged 50-69 years, <110 mmHg for others, requiring intervention other than reduction of esmolol dose at any point during study day; vasopressor infusion rates – micrograms/kilograms/minute
