# Supplementary material for: Early Intravenous Beta-Blockade with Esmolol in Adults with Severe Traumatic Brain Injury: A Phase 2a Intervention Design Study
Source: Neurocrit Care. 2024 Jun 28;41(3):1009–19. doi: 10.1007/s12028-024-02029-8 (PMC11599627; doi:10.1007/s12028-024-02029-8)
Supplement: Supplementary file 2 — Supplementary file2 (DOCX 21 kb) [file 12028_2024_2029_MOESM2_ESM.docx]

| 3c. Therapy Intensity Level (TIL) scores (/38) | | | | | | |
| --- | --- | --- | --- | --- | --- | --- |
|  | Study day 0 | Study day 1 | Study day 2 | Study day 3 | Study day 4 | Study days 0 – 4 |
| *Patients receiving esmolol (/n)* | *16* | *14* | *9* | *8* | *6* | *53* |
| TIL24, 8am-8am (median, IQR) | 5.5 (5.0 – 6.8) | 5.0 (4.3 – 6.8) | 5.0 (4.0 – 6.0) | 5.0 (4.8 – 5.3) | 5.5 (3.5 – 6.8) | 5.0 (5.0 – 6.0) |
| Domain TIL24 (median, IQR), /maximum |  |  |  |  |  |  |
| Head elevation, /1 | 1.0 (1.0 – 1.0) | 1.0 (1.0 – 1.0) | 1.0 (1.0 – 1.0) | 1.0 (1.0 – 1.0) | 1.0 (1.0 – 1.0) | 1.0 (1.0 – 1.0) |
| Sedation/Paralysis, /8 | 2.0 (1.0 – 2.0) | 1.5 (1.0 – 2.0) | 1.0 (1.0 – 2.0) | 1.0 (0.0 – 2.0) | 1.0 (0.3 – 2.0) | 2.0 (1.0 – 2.0) |
| CSF drainage, /3 | 0.0 (0.0 – 0.0) | 0.0 (0.0 – 0.0) | 0.0 (0.0 – 0.0) | 0.0 (0.0 – 1.5) | 0.0 (0.0 – 3.0) | 0.0 (0.0 – 0.0) |
| Fluid boluses/vasopressors, /2 | 2.0 (1.0 – 2.0) | 2.0 (1.0 – 2.0) | 1.0 (1.0 – 2.0) | 1.0 (1.0 – 2.0) | 1.0 (1.0 – 1.8) | 2.0 (1.0 – 2.0) |
| Hypocapnia intensity, /4 | 1.0 (0.0 – 1.0) | 1.0 (0.0 – 1.0) | 1.0 (1.0 – 1.0) | 1.0 (0.8 – 1.3) | 0.5 (0.0 – 1.0) | 1.0 (0.0 – 1.0) |
| Hyperosmolar therapy, /6 | 0.0 (0.0 – 0.0) | 0.0 (0.0 – 0.0) | 0.0 (0.0 – 0.0) | 0.0 (0.0 – 0.0) | 0.0 (0.0 – 0.0) | 0.0 (0.0 – 0.0) |
| Temperature management, /5 | 0.0 (0.0 – 0.3) | 0.0 (0.0 – 0.0) | 0.0 (0.0 – 0.0) | 0.0 (0.0 – 0.0) | 0.0 (0.0 – 0.0) | 0.0 (0.0 – 0.0) |
| Surgical intervention, /9 | 0.0 (0.0 – 0.0) | 0.0 (0.0 – 0.0) | 0.0 (0.0 – 0.0) | 0.0 (0.0 – 0.0) | 0.0 (0.0 – 0.0) | 0.0 (0.0 – 0.0) |
| Domain TIL24 score assignments (n/%) |  |  |  |  |  |  |
| Head elevation for ICP control | 16 (100) | 14 (100) | 9 (100) | 8 (100) | 6 (100) | 53 (100) |
| Sedation for ICP control (no paralysis)* | 8 (50) | 4 (29) | 3 (33) | 3 (37) | 3 (50) | 21 (40) |
| Sedation + paralysis for ICP control | 11 (69) | 7 (50) | 3 (33) | 3 (38) | 3 (50) | 27 (51) |
| EVD with CSF drainage (<120ml/day) | 1 (6) | 0 (0) | 0 (0) | 0 (0) | 0 (0) | 1 (2) |
| EVD with CSF drainage (>120ml/day) | 1 (6) | 2 (14) | 2 (22) | 2 (25) | 2 (33) | 2 (19) |
| Vasopressor infusion (any dose) | 16 (100) | 14 (100) | 9 (100) | 8 (100) | 6 (100) | 53 (100) |
| Fluid loading (any volume) | 11 (69) | 8 (57) | 4 (44) | 3 (38) | 2 (33) | 28 (53) |
| Fluid loading + vasopressor | 16 (100) | 14 (100) | 9 (100) | 8 (100) | 6 (100) | 53 (100) |
| Highest PaCO2 4.6-5.3 kPa | 9 (56) | 5 (36) | 5 (56) | 3 (38) | 2 (33) | 24 (45) |
| Highest PaCO2 4.0-4.5 kPa | 2 (13) | 0 (0) | 2 (22) | 2 (25) | 0 (0) | 6 (11) |
| Highest PaCO2 <4.0 kPa | 0 (0) | 2 (14) | 0 (0) | 0 (0) | 0 (0) | 2 (4) |
| Hypertonic 5% saline (<0.3g/kg/day)* | 2 (13) | 0 (0) | 0 (0) | 0 (0) | 1 (17) | 3 (6) |
| Cooling mattress (TTM)* | 4 (25) | 3 (21) | 0 (0) | 0 (0) | 0 (0) | 7 (13) |
| Surgical intervention | 0 (0) | 1 (7) | 0 (0) | 0 (0) | 0 (0) | 1 (2) |

* Sedation with higher-dose sedation or aim of metabolic suppression for intracranial pressure (ICP) control; no patients received mannitol or >0.3g/kg/day of 5% saline; TTM: targeted temperature management (prevention of temperature >38.0 and avoidance of hypothermia <36.0)
